# Influence of Stress and Antibiotic Resistance on Cell-Length Distribution in *Mycobacterium tuberculosis* Clinical Isolates

**DOI:** 10.3389/fmicb.2017.02296

**Published:** 2017-11-21

**Authors:** Srinivasan Vijay, Dao N. Vinh, Hoang T. Hai, Vu T. N. Ha, Vu T. M. Dung, Tran D. Dinh, Hoang N. Nhung, Trinh T. B. Tram, Bree B. Aldridge, Nguyen T. Hanh, Do D. A. Thu, Nguyen H. Phu, Guy E. Thwaites, Nguyen T. T. Thuong

**Affiliations:** ^1^Oxford University Clinical Research Unit, Ho Chi Minh City, Vietnam; ^2^Centre for Tropical Medicine and Global Health, Nuffield Department of Medicine, University of Oxford, Oxford, United Kingdom; ^3^Department of Molecular Biology and Microbiology, Tufts University School of Medicine, Boston, MA, United States; ^4^Department of Biomedical Engineering, Tufts University School of Engineering, Medford, MA, United States; ^5^Hospital for Tropical Diseases, Ho Chi Minh City, Vietnam

**Keywords:** *Mycobacterium tuberculosis*, cell-length variation, macrophage infection, sputum, multidrug resistance, oxidative stress, rifampicin, iron deficiency

## Abstract

Mycobacterial cellular variations in growth and division increase heterogeneity in cell length, possibly contributing to cell-to-cell variation in host and antibiotic stress tolerance. This may be one of the factors influencing *Mycobacterium tuberculosis* persistence to antibiotics. Tuberculosis (TB) is a major public health problem in developing countries, antibiotic persistence, and emergence of antibiotic resistance further complicates this problem. We wanted to investigate the factors influencing cell-length distribution in clinical *M. tuberculosis* strains. In parallel we examined *M. tuberculosis* cell-length distribution in a large set of clinical strains (*n* = 158) from *ex vivo* sputum samples, *in vitro* macrophage models, and *in vitro* cultures. Our aim was to understand the influence of clinically relevant factors such as host stresses, *M. tuberculosis* lineages, antibiotic resistance, antibiotic concentrations, and disease severity on the cell size distribution in clinical *M. tuberculosis* strains. Increased cell size and cell-to-cell variation in cell length were associated with bacteria in sputum and infected macrophages rather than liquid culture. Multidrug-resistant (MDR) strains displayed increased cell length heterogeneity compared to sensitive strains in infected macrophages and also during growth under rifampicin (RIF) treatment. Importantly, increased cell length was also associated with pulmonary TB disease severity. Supporting these findings, individual host stresses, such as oxidative stress and iron deficiency, increased cell-length heterogeneity of *M. tuberculosis* strains. In addition we also observed synergism between host stress and RIF treatment in increasing cell length in MDR-TB strains. This study has identified some clinical factors contributing to cell-length heterogeneity in clinical *M. tuberculosis* strains. The role of these cellular adaptations to host and antibiotic tolerance needs further investigation.

## Introduction

Tuberculosis (TB) remains as an important and difficult to treat human disease. Prolonged antimicrobial treatment is required to prevent clinical complications and cure the infection. Antimicrobial resistance further increases the risk of treatment failure and poor clinical outcomes (Stewart et al., 2003; Gomez and McKinney, 2004; Mitchison and Davies, 2012). In addition bacterial population heterogeneity generates antibiotic tolerant subpopulations which may contribute to clinical persistence (Balaban et al., 2004; Veening et al., 2008). Several recent studies have studied the complexity of persister sub-populations, revealing different mechanisms for generating antibiotic persistence, their role in treatment failure, and possible approaches to eradicating such persister populations (Van den Bergh et al., 2017). Recently bacterial antibiotic tolerance has also been implicated in the emergence of antibiotic-resistant strains (Levin-Reisman et al., 2017). Thus, the presence of such tolerant subpopulations in *Mycobacterium tuberculosis* may augment the clinical complications associated with TB.

In some studies mycobacterial cell length and elongation rates are associated with differential susceptibility to host- and antibiotic-induced stress (Aldridge et al., 2012; Richardson et al., 2016; Vijay et al., 2017). The exact mechanisms contributing to such stress tolerance is not clear. *M. smegmatis* divides asymmetrically, producing daughter cells with different characteristics. For example, the daughter cell which is shorter and elongates slower is more tolerant to cell wall inhibiting antibiotics (Aldridge et al., 2012) than its sister which is longer and elongates faster. On the other hand, the longer, faster-growing daughter is more tolerant to rifampicin (RIF), than the shorter, slower daughter at the early stages of cell division (Richardson et al., 2016). However, other studies have not observed differential susceptibility to antibiotics based on cell length and elongation rates (Santi et al., 2013). In *M. smegmatis* both short and long size resting cells were generated under different starvation models, and both the types of cells were found to be tolerant to antibiotics (Wu et al., 2016). In *M. smegmatis* sub-populations of cells were observed to grow, divide, and die during the persistence phase of isoniazid killing, and this was independent of single-cell growth rates (Wakamoto et al., 2013). It is also observed that cell size- and density-specific subpopulations exist in mycobacteria, and long cell size is associated with tolerance to host and antibiotic stress conditions (Vijay et al., 2017). Antibiotic tolerant cells can also give rise to antibiotic-resistant cells in *M. tuberculosis* during antibiotic treatment, possibly due to growth under antibiotic selection pressure (Wakamoto et al., 2013; Sebastian et al., 2017).

Mechanisms explaining heterogeneity in mycobacterial cell length are emerging from recent studies on mycobacterial cell biology (Kieser and Rubin, 2014). Asymmetric cell division is commonly observed in mycobacteria increasing cell-length heterogeneity in the population (Kieser and Rubin, 2014). Asymmetric cell division is due to mechanisms unique to mycobacteria which have only been partly clarified (Hett and Rubin, 2008). These include differential elongation rates of mycobacterial cell poles (Hett and Rubin, 2008; Aldridge et al., 2012; Kieser and Rubin, 2014), asymmetric localization of cell division proteins (Joyce et al., 2012; Singh et al., 2013), and asymmetric positioning of the septum toward the new cell pole and size-dependent growth, where the longer old-pole daughter elongates at faster velocity than its shorter new-pole sibling (Santi et al., 2013). It has also been observed that mycobacterial cells inheriting an old pole are able to elongate faster than the cells inheriting a new pole (Aldridge et al., 2012). Cells inheriting an old pole have longer cell length at birth and elongate faster compared to cells inheriting a new pole (Santi et al., 2013). Study of the distribution of irreversibly oxidized proteins (IOPs) in *M. smegmatis* and *M. tuberculosis* has revealed that the IOPs are associated with chaperone ClpB, and get asymmetrically distributed between progeny cells during cell division (Vaubourgeix et al., 2015). The progeny cells inheriting a higher level of IOPs grow slowly and are more susceptible to antibiotics (Vaubourgeix et al., 2015). In both *M. smegmatis* and *M. tuberculosis* segresomes asymmetry may also contribute to asymmetric growth and division of cells (Ginda et al., 2017). Supporting these observations deletion of mycobacterial cellular factor LamA involved in asymmetry in cell pole growth reduces both cell size heterogeneity and antibiotic tolerance in *M. smegmatis* and *M. tuberculosis* strains (Rego et al., 2017). Ultrastructural studies have revealed enhanced asymmetric cell division, and other cellular adaptations associated with multidrug resistance (Farnia et al., 2010). Hence, cell-length heterogeneity in mycobacterial population is established during cell elongation and division, generating long and short cells, contributing to antibiotic tolerance (Vijay et al., 2014a,b, 2017).

In addition, host stress factors may further increase the phenotypic heterogeneity in mycobacterial population. As observed in a murine *M. tuberculosis* infection model system, presence of host interferon-gamma results in a subpopulation of *M. tuberculosis* cells which can persist during isoniazid (INH) treatment (Manina et al., 2015). In this study increased *M. tuberculosis* cell length and heterogeneity in size distribution were also observed in wild-type mice compared to *in vitro* culture or in interferon-gamma-deficient mice (Manina et al., 2015). Host immune activation of macrophages also induces antibiotic tolerance in *M. tuberculosis* cells (Liu et al., 2016).

Thus, various studies suggest that cell-length heterogeneity exists in *M. tuberculosis* and possibly contributes to host and antibiotic stress tolerance. There is, however, limited knowledge regarding the manifestations of cell-length distribution in clinical *M. tuberculosis* strains, the impact of host stress on the cell-length distribution, and the clinical consequences. Most of the research conducted on mycobacterial cell size heterogeneity has used laboratory strains of mycobacteria, which may not represent the disease-causing strains freshly isolated from patients. In clinical conditions *M. tuberculosis* also grows and divides in human macrophages, evading macrophage-mediated killing. Thus, it is important to replicate the stress factors induced by macrophages on the bacteria when studying the consequences to the cell-length distribution.

We hypothesized that the host environment influences the cell-length distribution in clinical *M. tuberculosis* strains. In addition to the host stress, we also investigated the antibiotic-resistant phenotype which alters cellular physiology (Koch et al., 2014) and may also contribute to the cellular heterogeneity. We studied the cell-length distribution in 158 clinical *M. tuberculosis* strains including MDR strains, under different host stress conditions. We investigated *M. tuberculosis* cell-length distribution in sputum from pulmonary TB (PTB) patients. *M. tuberculosis* strains cultured from these sputum samples were examined for their cell-length distributions in a macrophage infection model, compared to their respective *in vitro* culture. We also investigated the associations of *M. tuberculosis* cell-length distribution with *M. tuberculosis* lineages, oxidative stress, iron deficiency, antibiotic-resistant strains, and TB severity in patients.

## Materials and Methods

### Bacterial Isolates

**FIGURE 1:**
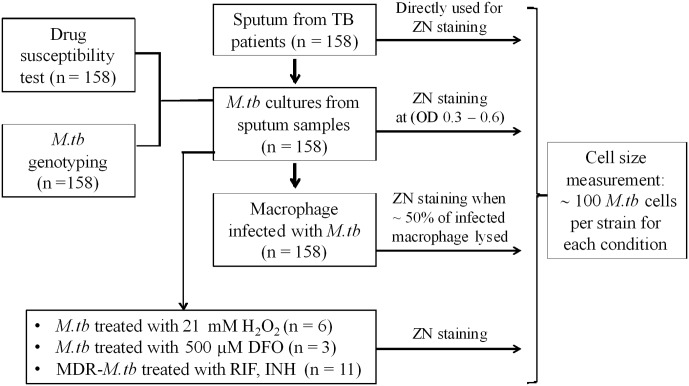
Study design.

*Mycobacterium tuberculosis* isolates used in this study (**Figure 1**) were collected from patients with PTB. Between January 2015 and October 2016, 158 PTB patients were recruited from two district TB control units in Ho Chi Minh City (HCMC), Vietnam. Patients were ≥18 years of age, had clinical symptoms of active PTB, which was confirmed by chest X-ray and positive sputum culture, and all were HIV uninfected.

### Ethics Statement

All patients recruited for the study were ≥18 years of age, written informed consent was obtained from each patient in accordance with the Declaration of Helsinki. Protocols were approved by the human subjects review committees, at the Hospital for Tropical Diseases HCMC, Vietnam (124/BVBNÐ.HÐÐÐ) and the Oxford Tropical Research Ethics Committee, United Kingdom (OxTREC Reference: 16-14). Baseline characteristics of patients were collected during the recruitment for the study.

### Sputum Collection and Bacterial Growth

Sputum was collected from 158 PTB patients, before initiation of anti-tuberculosis treatment, all the *M. tuberculosis* samples were handled in biosafety level-3 laboratory. All sputum samples were subject to Ziehl-Neelsen (ZN) staining and microscopy. *M. tuberculosis* strains were then isolated from sputum by culture in 7H9 liquid medium. These clinical strains were stored as glycerol stocks and were used for further experiments with limited number (∼2–3) of sub-culturing to avoid phenotypic/genotypic changes. For mid-log culture conditions, 50 ml culture tubes with 10 ml 7H9T medium [7H9 broth supplemented with 10% Oleic acid/Albumin/Dextrose/Catalase (OADC) enrichment, and 0.05% Tween 80, BD Difco^TM^] were inoculated with the clinical isolates and a laboratory strain H37Rv, incubated at 37°C with shaking. Samples for ZN staining and microscopy were performed at OD_600_ of 0.3–0.6.

*Mycobacterium tuberculosis* lineage identification: *M. tuber culosis* lineages were identified by large sequence polymorphism (LSP) typing (Gagneux et al., 2006). *M. tuberculosis* DNA was extracted from cultures on LJ media (Becton Dickinson, United States) by cetyltrimethyl ammonium bromide (CTAB) method. *M. tuberculosis* cells were dispersed in 600 μl TE buffer (10 mM Tris pH 8.0 and 1 mM EDTA), followed by heating the tube at 80°C for 20 min. Fifty microliters of lysozyme 10 mg/ml was added; the cell suspension was mixed well by vortexing and incubated overnight at 37°C. Next, 10 μl of proteinase K (10 mg/ml) and 35 μl of SDS 20% were added and incubated at 55°C for 30 min. Following this, to the suspension 100 μl 5 M NaCl and 100 μl 5% CTAB solution (5% CTAB and 0.5 M NaCl) were added and incubated at 60°C for 15 min. Two phases of DNA and protein-cell debris were separated by the addition of 700 μl chloroform and centrifugation at 12,000 × *g* for 5–10 min. The DNA phase was separated and precipitated with 0.6 volume of isopropanol by centrifugation at 13,000 × *g* for 15 min. DNA was washed with 70% ethanol, air-dried, and re-suspended in 40–80 μl TE buffer or distilled water.

Large sequence polymorphisms were then defined by the following method (Tsolaki et al., 2005; Caws et al., 2008); all the strains were first characterized for RD239 and RD105 deletion by PCR, as the majority of isolates were anticipated to contain one of these two deletions. Isolates bearing RD239 deletion were defined as Indo-Oceanic genotype while isolates bearing RD105 deletion were defined as East-Asia/Beijing genotype. The amplification using RD105 primers produced a product of 4 kb in the control H37Rv while it was 850 kb in isolates bearing RD105 deletion. Likewise, product from amplification using RD239 primers was 1.8 kb in control H37Rv and was shorter in isolates bearing RD239 deletion. Isolates without RD105 or RD239 were further defined for the Euro-American lineage by PCR to detect the deletion of 7 bp in the *pks* gene. Isolates bearing a deletion of 7 bp produced two bands of 520 and 259 bp whereas isolates without the deletion produced a single band of 520 bp.

### Macrophage Growth and Infection

J774 mouse macrophages (J774 murine cell line, J774A.1, ATCC TIB-67) were infected with the clinical *M. tuberculosis* strains and H37Rv. One day prior to infection, 24-well plates were seeded with 8 × 10^4^ J774 cells (measured from slides with grid chamber, KOVA Glasstic Slide) in 1 ml complete medium [RPMI 1640 medium plus 10% FBS, 1% L-glutamine, 1% penicillin, and streptomycin (STR), Sigma–Aldrich]. On the day of infection, old medium was removed and fresh pre-warmed infection medium (RPMI 1640 medium plus 10% FBS, 1% L-glutamine) was added. *M. tuberculosis* cultures at OD_600_ of 0.5–0.6 were vortexed with beads to remove the clumps, and this culture was diluted in pre-warmed uptake buffer to obtain a multiplicity of infection 4 (MOI) in macrophages. Plates were incubated at 37°C and 5% CO_2_. After 3 days post-infection, extracellular *M. tuberculosis* cells were washed off and the new infection medium was added. Infected macrophages were observed by light microscopy daily, to ascertain host cell lysis. When ∼50% of J774 cells were lysed (6–7 days post-infection), cells were collected by trypsinization and smears were prepared for ZN staining. For macrophage infection along with RIF treatment of MDR-TB strains, 24-well plates were seeded with 2.5 × 10^5^ THP1 cells (THP1 human cell line, 88081201, Sigma–Aldrich) (measured from slides with grid chamber, KOVA Glasstic Slide). THP1 cells were induced by 100 nM phorbol 12-myristate 13-acetate (PMA, Sigma–Aldrich) for 48 h and then grown in PMA-free complete medium for at least 24 h. Prior to the infection, *M. tuberculosis* cells grown in 7H9 medium to OD_600_ 0.5–0.8 were bead beaten to remove the clumps and then diluted to final volume of 1 ml in uptake buffer for THP1 infection at MOI = 1. Plates were incubated at 37°C and 5% CO_2_. After 4 h post-infection, extracellular *M. tuberculosis* cells were removed by washing and new infection medium was added. Twenty-four hours post-infection, infected THP1 macrophages were treated with the fresh medium containing RIF (Sigma–Aldrich) at a final concentration of 1 μg/ml representing 1× minimum inhibitory concentration (MIC). For RIF untreated control set the old medium was replaced with the fresh medium alone. Daily observation of cell lysis was performed with light microscopy. Once cell lysis reached ∼50% in both THP1 sets with and without RIF treatment, then they were harvested as described above.

### H_2_O_2_ Stress and Iron Deficiency

Liquid cultures of *M. tuberculosis* clinical strains and H37Rv in 7H9T medium at OD_600_ of 0.3–0.4 were treated with H_2_O_2_ (Merck) at a concentration of 21 mM for 48 h at 37°C, as described with slight modification in the concentration (Voskuil et al., 2011). *M. tuberculosis* smears were prepared from the treated and untreated control samples for ZN staining. For iron deficiency *M. tuberculosis* strains were grown in 7H9T medium containing an iron chelator, deferoxamine (DFO) mesylate salt (Sigma–Aldrich), at a final concentration of 500 μM (Pal et al., 2015) in 10 ml liquid medium in 50 ml culture tubes and incubated at 37°C. When the cultures reached OD_600_ 0.3–0.4, smears were prepared along with untreated control for ZN staining.

### Drug Susceptibility Test (DST)

Drug susceptibility test was performed using BACTEC^TM^ MGIT^TM^ 960 SIRE Kit (BD), according to the manufacturer guidelines. Drug susceptibility was tested for STR (1.0 μg/ml), INH (0.1 μg/ml), RIF (1.0 μg/ml), and ethambutol (EMB, 5.0 μg/ml).

### Rifampicin and Isoniazid Treatment

Multidrug-resistant-tuberculosis strains were grown on LJ medium and colonies were scraped and re-suspended in 15 ml Falcon tube containing 5 ml 0.9% NaCl, 0.2% Tween 80, and 10 glass beads. The tube was vortexed and absorbance of suspension was measured at OD_600_ and turbidity was adjusted to 0.08–0.1 OD (equivalent to 0.5 McFarland). Hundred microliters of the suspension was transferred into a tube containing 10 ml of 7H9T broth supplemented with 10% OADC, to give an inoculum of ∼1 × 10^5^ cfu/ml. From this inoculum, 100 μl was dispensed into each well of UKMYC3 Sensititre Plates (Thermo scientific) containing different concentrations of RIF and INH. The plates were incubated for 14 days at 37°C and *M. tuberculosis* cells grown in the wells were harvested for ZN smears.

### Cell-Length Measurements and Statistical Analysis

Ziehl-Neelsen stained slides were observed by light microscopy (Olympus) with a 100× oil immersion objective. Images were acquired using a DP21 Olympus camera and cellScens imaging software (Olympus). Cell-length measurements were carried out using ImageJ (Schneider et al., 2012), individual cell lengths were measured manually using segmented line in ImageJ by tracking along the cell as the sum of short linear segments (Santi et al., 2013). About 100 cell-length measurements were taken in each *M. tuberculosis* strain under each growth condition. To evaluate the cell size heterogeneity of *M. tuberculosis* strains in different environments, values of mean, standard deviation (SD), and coefficient of variation (CV, defined by the SD divided by the mean) were employed. We either combined cell-length measurements from all *M. tuberculosis* strains or represented individual *M. tuberculosis* strains under a specific growth condition or group in the cell size heterogeneity analysis. We presented *M. tuberculosis* cell-length distribution with a 0.02 μm window of cell size histogram. Furthermore, we perform maximum-likelihood estimation for the observed *M. tuberculosis* cell length with the assumption that *M. tuberculosis* cell length has log normal distribution. The estimates are shown in Supplementary Table S1. To directly compare *M. tuberculosis* cell size among strains and different growth conditions, we grouped individual *M. tuberculosis* strains in sputum based on mean and SD of cell size using convex hull (where the convex hull of a data set is defined as minimal convex set that contains all of its data points). To understand adaptations of *M. tuberculosis* clinical isolates in different environments at the population level, we collected a large number of *M. tuberculosis* cells and measured the length of more than 45,000 cells (158 strains × 100 cells per isolate × 3 in sputum samples, infected macrophages, and cultures). We applied a non-parametric comparative distribution test (e.g., Kruskal–Wallis) and it gave very small *P*-values (<10^-10^) in all comparisons between different environments. To focus on biological significance, instead of using the Kruskal–Wallis test, we used the Bootstrap method and reported the confidence interval (CI) values. To determine the extent of cell-length variation or effect size between different growth conditions we measured the differences in the mean cell length between groups by using a bootstrapping method (Nakagawa and Cuthill, 2007). We fixed the number of iteration at 1000 and subsample size at 300. At each iteration, we recorded the difference in mean cell length between groups. In our analysis, we used 300 cells for subsample size because we performed the test with 250, 300, and 400 cells and we did not observe any significant difference between them. The 95% CI is defined by the 2.5 to 97.5% quantile. When difference in mean cell length and 95% CI for a group does not include zero then there is a significant difference in mean cell length between that group and control group (Nakagawa and Cuthill, 2007). All analyses and plots generation were implemented in Matlab R2013a (Georgescu et al., 2012). For individual *M. tuberculosis* strains, *P*-values of cell size distribution were determined by using ANOVA for comparison of more than two groups, and Mann–Whitney to compare two groups in GraphPad Prism software.

## Results

### *M. tuberculosis* Cell-Length Distribution in Sputum, Macrophage Infection, and Liquid Culture

**FIGURE 2:**
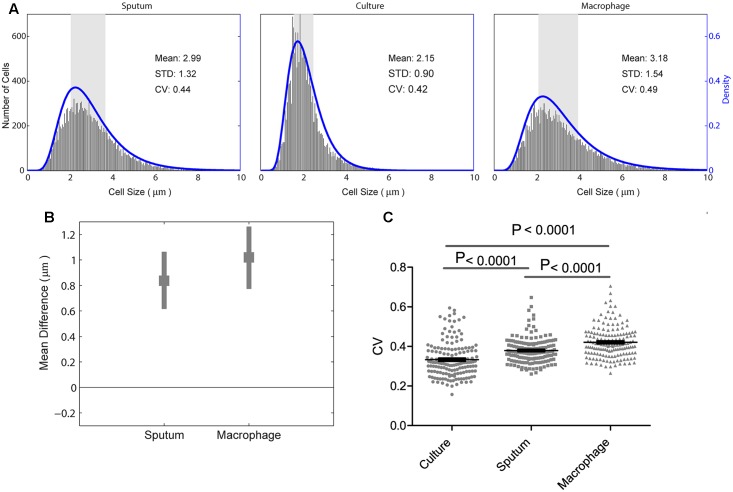
*Mycobacterium tuberculosis* cell-length distribution and CV between sputum, macrophage infection, and culture. **(A)** Absolute number of *M. tuberculosis* cell-length distribution (corresponding to right *y*-axis) combined from all the strains in sputum, culture, and infected macrophages (macrophage) are shown. The blue lines (corresponding to the right *y*-axis) are maximum-likelihood estimation for *M. tuberculosis* cell-length distribution. The gray rectangle represents the interquartile range of cell length. **(B)** Difference in mean *M. tuberculosis* cell length was compared between liquid culture and host conditions such as sputum and infected macrophages. Average mean difference (gray square dots) and 95% confidence interval (CI) (gray bars) of cell length in sputum, infected macrophages compared to liquid culture (base line corresponding to zero) by bootstrap method. If the average mean difference and 95% CI does not include zero for a group, then there is a significant difference in the mean cell length between that group and the base line. **(C)** Individual *M. tuberculosis* strains CV in culture, sputum, and infected macrophages, *P*-values by ANOVA, and Mann–Whitney test. SD, standard deviation; CV, coefficient of variation.

We analyzed *M. tuberculosis* cell-length distribution in ZN stained smears directly from the sputum samples from 158 untreated PTB patients. Cell-length distribution of *M. tuberculosis* strains isolated from these sputum samples were also analyzed in infected macrophages, and in their corresponding liquid cultures (**Figure 1**). This allowed us to determine and compare the extent of *M. tuberculosis* cell-length distribution in both intracellular and extracellular host environments (**Figure 2** and Supplementary Figure S1). To quantify *M. tuberculosis* cell-length distribution in different growth conditions, we analyzed the mean, SD, and CV of *M. tuberculosis* cell-length distribution and compared these values between different growth conditions. Cell-length measurements from clinical *M. tuberculosis* strains were combined (*n* ∼158 × 100) in each growth condition and compared between host conditions and liquid culture (**Figure 2A**). *M. tuberculosis* cells under host conditions had a wider range of cell lengths, with a CV (mean and SD) of 0.44 (3.0 ± 1.3 μm) in sputum and 0.49 (3.2 ± 1.5 μm) under macrophage infection (**Figure 2A**), compared with the narrow cell-length distribution seen in liquid culture 0.42 (2.2 ± 0.9 μm) (**Figure 2A**). As the cell lengths of *M. tuberculosis* strains were increased in host conditions than in culture, we further analyzed the differences in mean cell length between different conditions. This revealed the extent of difference in the cell-length distribution of *M. tuberculosis* between culture versus sputum or macrophage infection (**Figure 2B**). *M. tuberculosis* cell length increased in sputum and macrophage infection compared to liquid culture, with mean cell-length differences of 0.8 μm (95% CI = 0.63–1.06) in sputum and 1.0 μm (95% CI = 0.78–1.28) in macrophage infection (**Figure 2B**). Indicating that, increased cell length in *M. tuberculosis* is associated with host conditions compared to liquid culture.

Individual *M. tuberculosis* strains displayed a wide range of CVs from 0.2 to 0.7 in different growth conditions (**Figure 2C**) and increased CV under host conditions like sputum and macrophage infection than culture (*P* < 0.0001) (**Figure 2C**). To investigate if there is any correlation in the cell length of individual *M. tuberculosis* strains between different growth conditions, we plotted the mean and SD of each strain (*n* = 158, Supplementary Figure S2). We observed that the individual *M. tuberculosis* strains cell length can be divided into three groups as short, medium, and long in sputum based on their mean and SD of cell-length distribution (Supplementary Figure S2). From these three groups, individual *M. tuberculosis* strains cell length was correlated with their respective cell length under macrophage infection and in culture (Supplementary Figure S2). Individual *M. tuberculosis* strains in these three cell length groups from sputum underwent significant redistribution, as observed by the change in their respective mean and SD of cell length under macrophage infection and culture (Supplementary Figure S2). Indicating that individual clinical *M. tuberculosis* strains display a wide range of cell-length distribution, and for a given *M. tuberculosis* strain, cell-length distribution in one condition does not correlate with the distribution seen in another growth condition. Whereas by combining their cell lengths in each condition, we can investigate the overall association of cell length with different growth conditions.

### The Cell-Length Distribution of *M. tuberculosis* Strains Is Independent of Lineage

To measure lineage-specific correlation with *M. tuberculosis* cell-length distribution, we analyzed the cell-length distribution of major *M. tuberculosis* lineages prevalent in Vietnam, under host-like growth conditions including sputum and macrophage infection as well as in nutrient rich growth medium. We observed that there was no significant difference in the cell lengths between Euro-American or Indo-Oceanic versus East Asian (Beijing) lineage in sputum with mean cell-length differences of 0.0 and 0.3 μm (95% CI = -0.25 to 0.22 and 0.11–0.63), in culture -0.1 and 0.1 μm (95% CI -0.31 to -0.01 and -0.04 to 0.31) and under macrophage infection 0.1 and 0.0 μm (95% CI -0.17 to 0.43 and -0.30 to 0.28), respectively (Supplementary Figure S3A). The CV (mean and SD) of cell-length distribution combined from all *M. tuberculosis* strains in each of East-Asian (Beijing), Euro-American, and Indo-Oceanic *M. tuberculosis* lineages was determined under sputum [0.44 (2.8 ± 1.2 μm), 0.42 (2.8 ± 1.2 μm), and 0.43 (3.2 ± 1.3 μm)], culture [0.39 (2.1 ± 0.8 μm), 0.39 (1.9 ± 0.7 μm), and 0.43 (2.2 ± 0.9 μm)], and macrophage infection [0.48 (3.1 ± 1.5 μm), 0.51 (3.2 ± 1.6 μm), and 0.46 (3.1 ± 1.4 μm)], respectively (Supplementary Figure S3B). Indicating that mean cell length did not show any significant increase between different lineages, but in each lineage we also observed increased *M. tuberculosis* cell length under host conditions like sputum and macrophage infection compared to culture (Supplementary Figure S3B). Thus, *M. tuberculosis* cell-length distribution is independent of *M. tuberculosis* lineages, but strongly dependent on growth conditions like host conditions and liquid culture.

### Drug Resistance Increased *M. tuberculosis* Cell Length in Sputum and Macrophage Infection Compared to Culture

**FIGURE 3:**
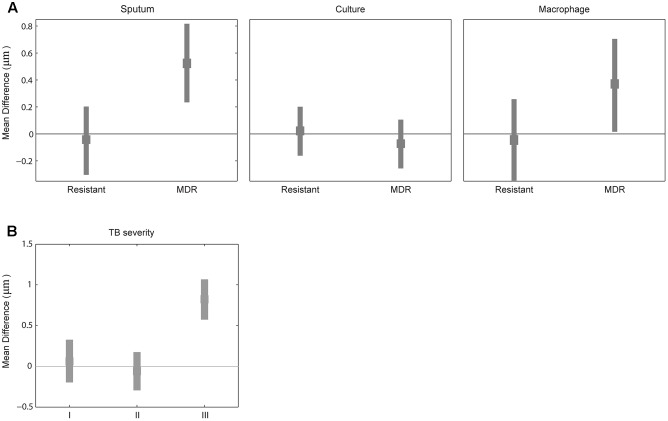
Difference in mean cell length between sensitive and resistant strains or by TB severity. **(A)** Difference in mean *M. tuberculosis* cell length between sensitive (line corresponding to zero) and other two (resistant and MDR) *M. tuberculosis* strains from sputum, culture, and macrophage. **(B)** Difference in mean *M. tuberculosis* cell length between patients sputum without blood and cavitary lesions (base line corresponding to zero) and different TB severity (I) with bloody sputum and without cavitary lesions, (II) without bloody sputum but with cavitary lesions, and (III) with both bloody sputum and cavitary lesions by bootstrap method.

Because mycobacterial cell length may be one of the factors contributing to drug tolerance in laboratory strains of antibiotic sensitive mycobacteria (Richardson et al., 2016; Vijay et al., 2017), we next examined whether MDR strains have different cell-length distributions than drug-sensitive strains by analyzing the cell-length distribution of drug-sensitive and -resistant *M. tuberculosis* strains. We measured drug susceptibility in each strain to STR, INH, RIF, and EMB. Using these drug resistance profiles we grouped *M. tuberculosis* strains as (1) susceptible to all four drugs (*n* = 72), (2) resistant to at least one drug but not to INH and RIF (*n* = 75), and (3) MDR resistant to at least to INH and RIF (*n* = 11). MDR strains displayed increased cell lengths in sputum with mean cell-length differences of 0.5 μm (95% CI = 0.25–0.81) and in macrophage infection 0.3 μm (95% CI = 0.05–0.70) compared to sensitive strains (**Figure 3A** and Supplementary Figure S1B). Whereas other resistant strains did not show any such significant difference in cell length in sputum with mean cell-length differences of 0.0 μm (95% CI = -0.30 to 0.22) and in macrophage infection 0.0 μm (95% CI = -0.33 to 0.25) compared to sensitive strains (**Figure 3A**). Further under liquid culture condition no such significant difference in cell length was observed between resistant and MDR strains with the mean cell-length differences of 0.0 and -0.0 μm (95% CI = -0.14 to 0.21 and -0.24 to 0.08), respectively, compared to sensitive strains (**Figure 3A**). The CV (mean and SD) of cell size distribution combined from all *M. tuberculosis* strains in each of sensitive, resistant, and MDR groups was determined under sputum [0.43 (3.0 ± 1.3), 0.45 (3.0 ± 1.4), and 0.45 (3.5 ± 1.6)], culture [0.42(2.2 ± 0.9), 0.42 (2.2 ± 0.9), and 0.43 (2.1 ± 0.9)], and macrophage infection [0.48 (3.2 ± 1.5), 0.47 (3.2 ± 1.5), and 0.47(3.5 ± 1.7)], respectively (Supplementary Figure S4A). Thus, MDR strains displayed increased cell lengths under host conditions like sputum and macrophage infection compared to sensitive and other resistant strains. Indicating that host stress and antibiotic resistance phenotype can increase the cell lengths of clinical *M. tuberculosis* strains.

### MDR-TB and Severe Tuberculosis Patients with Bloody Sputum and Lung Cavitary Lesions Exhibit Increased *M. tuberculosis* Cell Length

We classified tuberculosis patients based on clinical severity at the beginning of treatment into four groups: subjects with non-bloody sputum and cavitary lesions on chest X-ray (*n* = 111 cases including five MDR-TB), those with bloody sputum and no cavitary lesions (*n* = 10 cases including two MDR-TB); those without bloody sputum and with cavitary lesions (11 cases without any MDR-TB); and with bloody sputum and cavitary lesions (three cases including two MDR-TB). We observed increased *M. tuberculosis* cell length in sputum from patients with bloody sputum and cavitary lesions (Supplementary Figures S1C,D), with a mean cell-length difference of 0.8 μm (95% CI = 0.56–1.07) compared to baseline and other two clinical severities groups with mean cell-length differences of 0.0 μm (95% CI = -0.18 to 0.33) and 0.0 μm (95% CI = -0.28 to 0.19) (**Figure 3B**). We also observed that patients with both bloody sputum and cavitary lesions displayed increased mean cell-length distribution (3.7 μm ± 1.4) compared to other three groups (2.9 μm ± 1.3, 2.8 μm ± 1.3 and 2.9 μm ± 1.4) (Supplementary Figure S4B). Although we had very few severe TB cases analyzed in our study (*n* = 3), it indicated that severe TB conditions may be associated with increased *M. tuberculosis* cell-length distribution. This needs to be further validated by analyzing more patients with severe TB.

### Oxidative Stress and Iron Deficiency Increased Cell-Length Distribution in Clinical *M. tuberculosis* Strains

**FIGURE 4:**
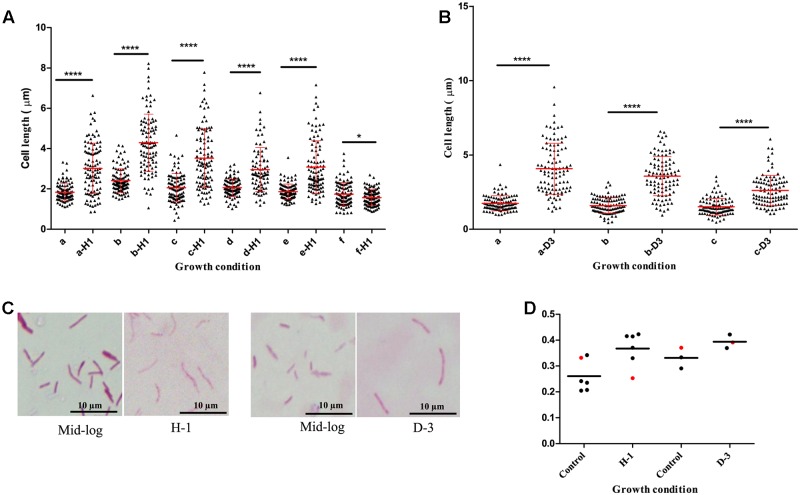
*Mycobacterium tuberculosis* cell-length distribution in individual strains under oxidative stress and iron deficiency. **(A)** *M. tuberculosis* cell-length distribution under oxidative stress (a-H1 to e-H1) five clinical *M. tuberculosis* strains and (f-H1) laboratory strain H37Rv treated with 21 mM H_2_O_2_ along with respective untreated mid-log controls (a–f). **(B)** *M. tuberculosis* cell-length distribution under iron deficiency (a-D3 and b-D3) two clinical *M. tuberculosis* strains, and (c-D3) laboratory strain H37Rv treated with 500 μM DFO along with respective untreated mid-log controls (a–c). In each strain *n* = 100 cell length in each condition. *P*-values by Mann–Whitney *U*-test, ^∗^*P* < 0.05, ^∗∗^*P* < 0.01, ^∗∗∗^*P* < 0.001, and ^∗∗∗∗^*P* < 0.0001. **(C)** ZN staining of a clinical *M. tuberculosis* strain from oxidative stress (H-1) and iron deficiency (D-3) along with mid-log controls. **(D)** CV of *M. tuberculosis* strains from oxidative stress (H-1) and iron deficiency (D-3), black circle indicates clinical *M. tuberculosis* strains and red circle represents H37Rv. DFO, deferoxamine mesylate salt.

We found host environments such as sputum and macrophage infection were associated with increased cell length in *M. tuberculosis* strains; we next investigated the influence of specific host factors on *M. tuberculosis* cell-length distribution. The mid-log culture of five clinical *M. tuberculosis* strains and H37Rv was treated with 21 mM concentration of H_2_O_2_ for oxidative stress and cell length was measured. We observed that H_2_O_2_ treatment resulted in significant increase of cell-length distribution and CV in clinical *M. tuberculosis* strains compared to untreated control (**Figures 4A,C,D**). But lab strain H37Rv in response to H_2_O_2_ treatment did not show such an increase in cell-length distribution and CV compared to clinical *M. tuberculosis* strains (**Figures 4A,D**). Similarly, clinical *M. tuberculosis* strains grown in the presence of iron deficiency induced by DFO mesylate salt at 500 μM concentration displayed increase in cell-length distribution and CV (**Figures 4B–D**); H37Rv grown in the presence of iron deficiency also displayed increase in cell-length distribution and CV, but to a lesser extent compared to clinical *M. tuberculosis* strains (**Figures 4B,D**). These results illustrate that host stress factors such as oxidative stress and iron deficiency can induce increased cell-length distribution and CV in *M. tuberculosis*, similar to that seen under sputum and macrophage infection.

### Rifampicin Treatment Increased Cell-Length Distribution in MDR-TB Strains

**FIGURE 5:**
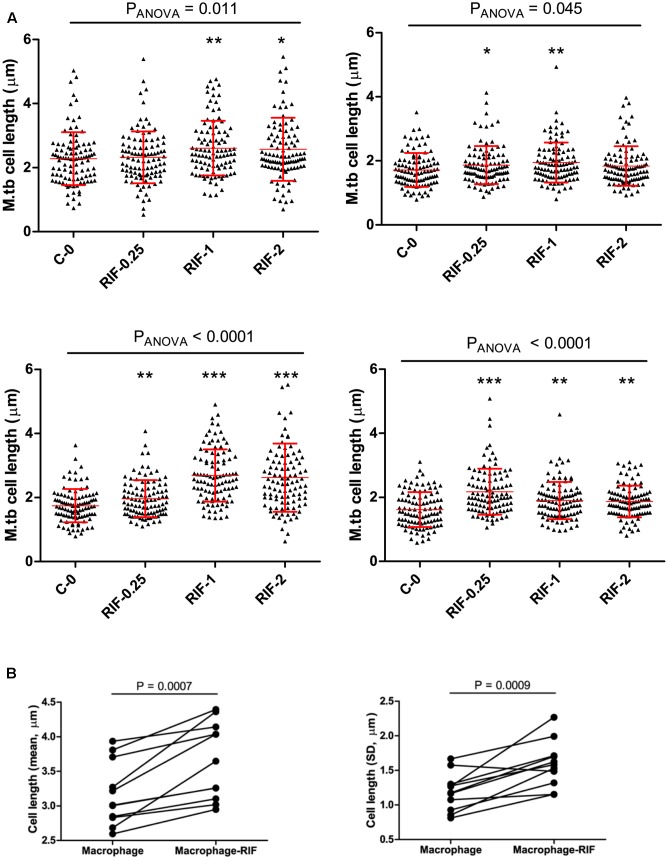
Cell length of MDR-TB strains under rifampicin and macrophage infection with Rif treatment. **(A)** MDR-TB strains (*n* = 4) cell-length distribution under different concentrations of RIF (μg/ml), MIC = 1.0 μg/ml, C-0 indicates no Rif. Comparisons across multiple groups of RIF concentrations were performed by one-way analysis of variance (ANOVA), comparisons of two groups by Mann–Whitney test. *P*-values by Mann–Whitney *U*-test between C-0 and RIF treatment, ^∗^*P* < 0.05, ^∗∗^*P* < 0.01, and ^∗∗∗^*P* < 0.001. **(B)** Mean and standard deviation (SD) of cell lengths of MDR-TB strains (*n* = 11) under macrophage infection and macrophage infection with RIF treatment at 1 μg/ml. Around 100 cells were measured in each MDR-TB strains. *P*-values by Mann–Whitney test.

To probe the influence of antibiotic treatment on the cell-length distribution in *M. tuberculosis* strains, we cultured selected clinical MDR-TB strains with below MIC, MIC, and above MIC concentrations of RIF and INH (**Figure 5A** and Supplementary Figure S5). Since MDR-TB strains can grow and divide in the presence of these antibiotics and exhibit the influence of RIF and INH treatment on their cell-length distribution. When four individual MDR-TB strains were cultured under different concentrations of RIF and INH (**Figure 5A** and Supplementary Figure S5), we observed that treatment with different RIF concentrations significantly (*P* ≤ 0.0001, ANOVA) increased cell-length distributions in two of the MDR-TB strains compared to respective untreated control (**Figure 5A**). In other two MDR-TB strains, only moderate increase in the cell-length distributions was observed (*P* = 0.01 and 0.40, ANOVA) (**Figure 5A**). For three of the MDR-TB strains treated with different concentrations of INH, we observed only moderate increases in cell length compared to untreated control (*P* = 0.001, 0.002, and 0.16) (Supplementary Figure S5). But one MDR-TB strain grown under different concentrations of INH showed significant increase in cell-length distribution (*P* < 0.0001, ANOVA) (Supplementary Figure S5). These results indicate a possible role of RIF treatment in increasing cell-length distribution in MDR-TB strains.

### Rifampicin and Macrophage Infection Synergistically Increase Cell-Length Distributions in MDR-TB Strains

Because we observed that both host stress conditions and RIF treatment enhanced cell length in MDR-TB strains, we next investigated is there any synergistic effect between the two conditions in increasing cell length in MDR-TB strains. Individual MDR-TB strain cell length in macrophage infection was compared with cell length in macrophage infection along with RIF treatment at 1 μg/ml concentration (**Figure 5B**). The results indicated that macrophage infection along with RIF treatment displayed synergism in increasing the cell lengths of MDR-TB strains than macrophage infection alone in MDR-TB strains (*n* = 11, *P* = 0.0007 and 0.0009) (**Figure 5B**).

## Discussion

*Mycobacterium tuberculosis* cell-length variations may be one of the factors influencing tolerance to host and antibiotics stress (Richardson et al., 2016; Vijay et al., 2017). We hypothesized that the host factors and antibiotic-resistant phenotype can influence *M. tuberculosis* cell-length distribution. Using an extensive analysis of cell-length variation in a large number of clinical isolates, we found that host conditions such as sputum and macrophage infection display increased *M. tuberculosis* cell length compared to *in vitro* culture. Under host conditions, host stress may be associated with increased cell length in *M. tuberculosis* strains. Oxidative stress and iron deficiency are some of the prominent host stresses encountered by *M. tuberculosis* during its growth inside the host (Lee et al., 2008; Chawla et al., 2012; Wells et al., 2013). We analyzed *M. tuberculosis* cell-length distribution under these host stress conditions and found increased cell-length distribution in *M. tuberculosis* strains. The results indicate that host stress is associated with increased cell length in *M. tuberculosis* strains as also observed in *M. tuberculosis* infection in mice (Manina et al., 2015). In the laboratory strain H37Rv we did not observe a similar increase in cell-length distribution as measured in clinical *M. tuberculosis* strains, indicating possible differential regulation of cellular processes in clinical *M. tuberculosis* strains under host stress conditions, as clinical *M. tuberculosis* strains are adapted to the host environment better than laboratory strains.

Other factors contributing to increased cell-length distribution were MDR/RIF-resistant phenotype under host conditions and during growth under RIF treatment. In addition to MDR-TB, there may also be RIF and INH heteroresistance where subpopulations of RIF- and INH-resistant *M. tuberculosis* cells can coexist with predominantly sensitive strains as observed in TB patients (Hofmann-Thiel et al., 2009). Both MDR-TB strains under host conditions and RIF heteroresistance under RIF treatment may contribute to increasing *M. tuberculosis* cell-length distribution in patients. INH treatment of sensitive *M. tuberculosis* strains *in vitro* resulted in cell size reduction (Manina et al., 2015), whereas MDR-TB strains cultured under INH did not exhibit any cell size reduction. Further we have observed synergistic increase in cell-length distribution among MDR-TB strains under macrophage infection along with RIF treatment. Supporting these observations, *M. tuberculosis* infection of macrophages along with anti-TB drugs treatment increased redox heterogeneity in both sensitive and resistant clinical isolates. Redox heterogeneity in *M. tuberculosis* cells also generates sub-populations with differential susceptibility to antibiotics (Bhaskar et al., 2014). Another factor influencing growth rate of *M. tuberculosis* during macrophage infection was MOI of infection. As low and high MOI of *M. tuberculosis* infection of macrophages resulted in different intracellular growth rate but similar level of antibiotic susceptibility (Raffetseder et al., 2014). The link between MOI of infection, growth rate, and cell-length distribution of *M. tuberculosis* during macrophage infection needs further investigation. Clinically, we also observed increased *M. tuberculosis* cell-length distribution in sputum samples from patients with severe form of TB, having both blood in sputum and lung cavitary lesions compared to patients lacking such severe TB symptoms. These observations reveal that *M. tuberculosis* strains exhibit increased cell-length distribution under diverse clinically associated conditions.

Evidence for the advantages of such cellular adaptations for the emergence of stress-tolerant subpopulations is emerging in microorganisms. Recent studies in *Escherichia coli* have shown differential partitioning of efflux pumps between parental and daughter cells, depending on the age of cell pole in rod-shaped bacteria, leading to cell-to-cell variations in antibiotic tolerance (Bergmiller et al., 2017). Asymmetric distribution of IOPs in progeny cells of mycobacteria also leads to differential susceptibility to antibiotics (Vaubourgeix et al., 2015). It has also been observed that there is difference in the density of membrane vesicles between long and short cells in mycobacteria (Vijay et al., 2017). These observations indicate that emergence and increase in cell-to-cell variations facilitate differential susceptibility to stress conditions in bacteria.

Currently, we have limited understanding about the influence of host factors on the cell elongation and division of *M. tuberculosis* cells. Recently, it has been observed that changing to less optimal carbon sources results in reduced cell elongation rate and increased interdivision time resulting in mean cell size differences in *M. smegmatis* cells (Priestman et al., 2017). Our study shows that host factors such as oxidative stress and iron deficiency may influence cell growth and division in *M. tuberculosis* strains, their influence on *M. tuberculosis* cell biology needs to be investigated further in detail. Host stress and antibiotic resistance may also induce stringent response in *M. tuberculosis* cells, which may contribute to increased cell-length distribution. It has been shown that in mycobacteria, modified nucleotide (p)ppGpp is synthesized by Rel enzyme during stringent response under starvation (Avarbock et al., 2005), which acts as a bacterial alarmone, and enables to regulate cell size and morphogenesis (Gupta et al., 2016; Wu et al., 2016). Interestingly, RIF resistance is also associated with induction of (p)ppGpp synthesis as an adaptation associated with antibiotic resistance (Koch et al., 2014). In our study, cell-length distribution was increased in MDR strains, and the role of (p)ppGpp in cell size regulation of MDR strains needs further investigation. Recently, mycobacterial cellular factor LamA has been shown to regulate differential polar growth which also generates cellular heterogeneity and differential susceptibility to antibiotics (Rego et al., 2017). It will be interesting to investigate how host factors influence such mycobacterial cellular factors and regulate the cell-length distribution in *M. tuberculosis*.

The ability of *M. tuberculosis* cells to persist in the host during antibiotic treatment and the emergence of MDR-TB are currently major clinical complications associated with TB treatment. In this study, we show that *M. tuberculosis* cell-length distribution in clinical strains is closely associated with host factors. Host factors like oxidative stress is regulated by tumor necrosis factor (TNF), as TNF regulates mitochondrial reactive oxygen species from infected macrophages (Roca and Ramakrishnan, 2013). TNF is transcriptionally regulated by factors like *LTA4H* gene, which encodes enzymes involved in the synthesis of leukotriene B4 and its polymorphism in the human population is implicated in susceptibility and resistance to TB infection (Tobin et al., 2010). Thus, host factors variation in humans can also be a potential source of variations in cell-length distribution in clinical *M. tuberculosis* strains.

Further detailed investigation of these cellular adaptions and their variations among clinical and MDR *M. tuberculosis* strains are required and their mechanisms need to be delineated. These adaptions also need to be correlated with antibiotic tolerance and persistence so that their consequences for tuberculosis treatment can be determined.

## Author Contributions

SV, NT, NP, and GT conceived and designed the experiments. SV, HH, and DT did the experiments and SV, HH, DT, VH, VD, TD, HN, TT, and NH collected the data. DV, SV, GT, BA, and NT analyzed and interpreted the data. SV, HH, DT, VH, VD, TD, HN, TT, NH, NP, NT, GT, and BA drafted, revised the manuscript, and approved the final version.

## Conflict of Interest Statement

The authors declare that the research was conducted in the absence of any commercial or financial relationships that could be construed as a potential conflict of interest.
