# Supplementary material for: Influence of Stress and Antibiotic Resistance on Cell-Length Distribution in Mycobacterium tuberculosis Clinical Isolates
Source: Front Microbiol. 2017 Nov 21;8:2296. doi: 10.3389/fmicb.2017.02296 (PMC5702322; doi:10.3389/fmicb.2017.02296)
Supplement: Supplementary file 1 [file Table_1.docx]

**Table S1. Parameters used for maximum likelihood estimation for *M. tuberculosis* cell length distribution.**

| Growth environment | Parameter of log normal distribution | |
| --- | --- | --- |
|  | *µ* | σ^2^ |
| Culture | 0.69986 | 0.36032 |
| Sputum | 1.0045 | 0.43316 |
| Macrophage infection | 1.051 | 0.46607 |
