# Supplementary figures and images for: Influence of Stress and Antibiotic Resistance on Cell-Length Distribution in Mycobacterium tuberculosis Clinical Isolates
Source: Front Microbiol. 2017 Nov 21;8:2296. doi: 10.3389/fmicb.2017.02296 (PMC5702322; doi:10.3389/fmicb.2017.02296)

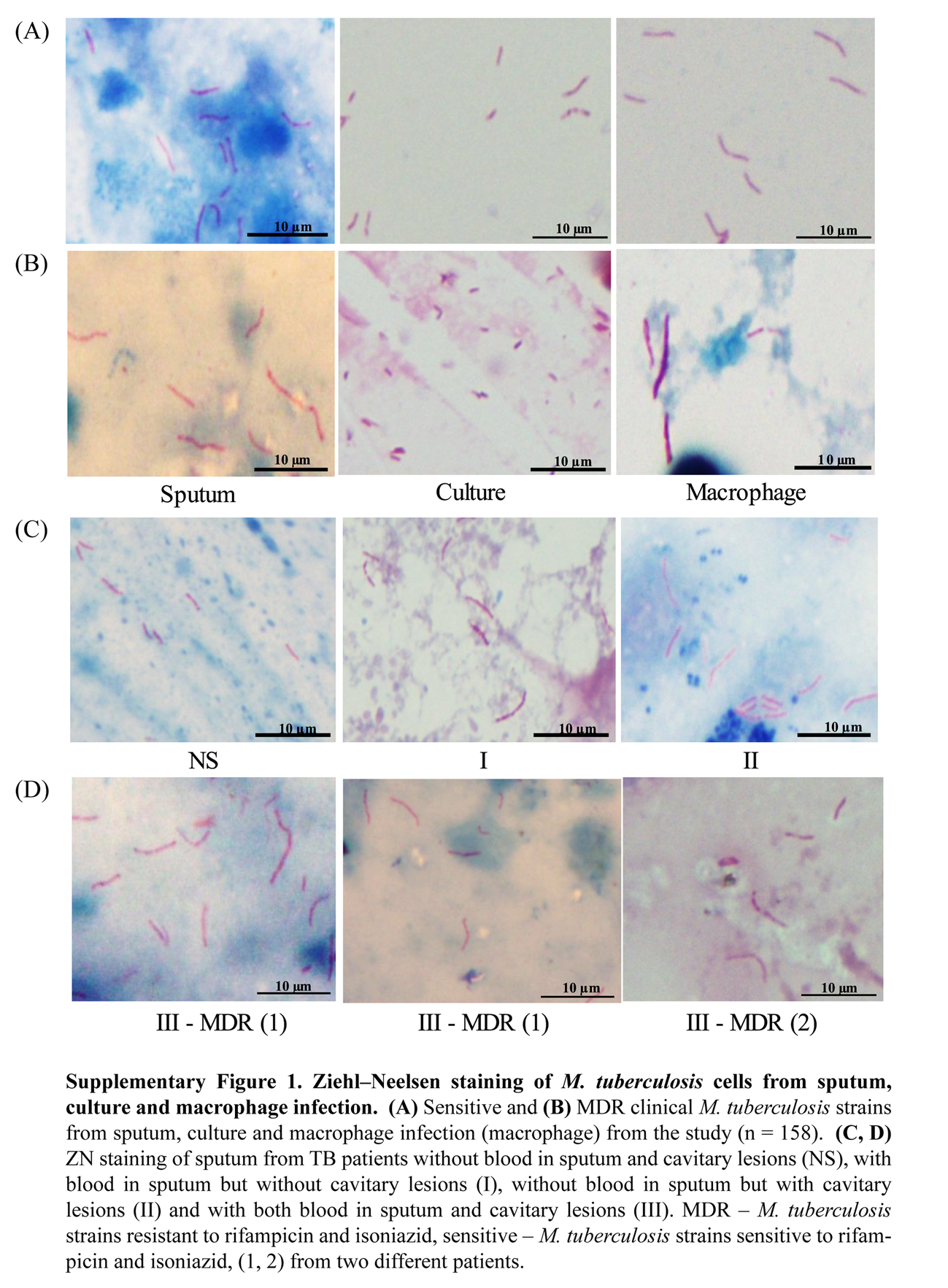

Supplement: Supplementary file 2 [file Image_1.TIF]

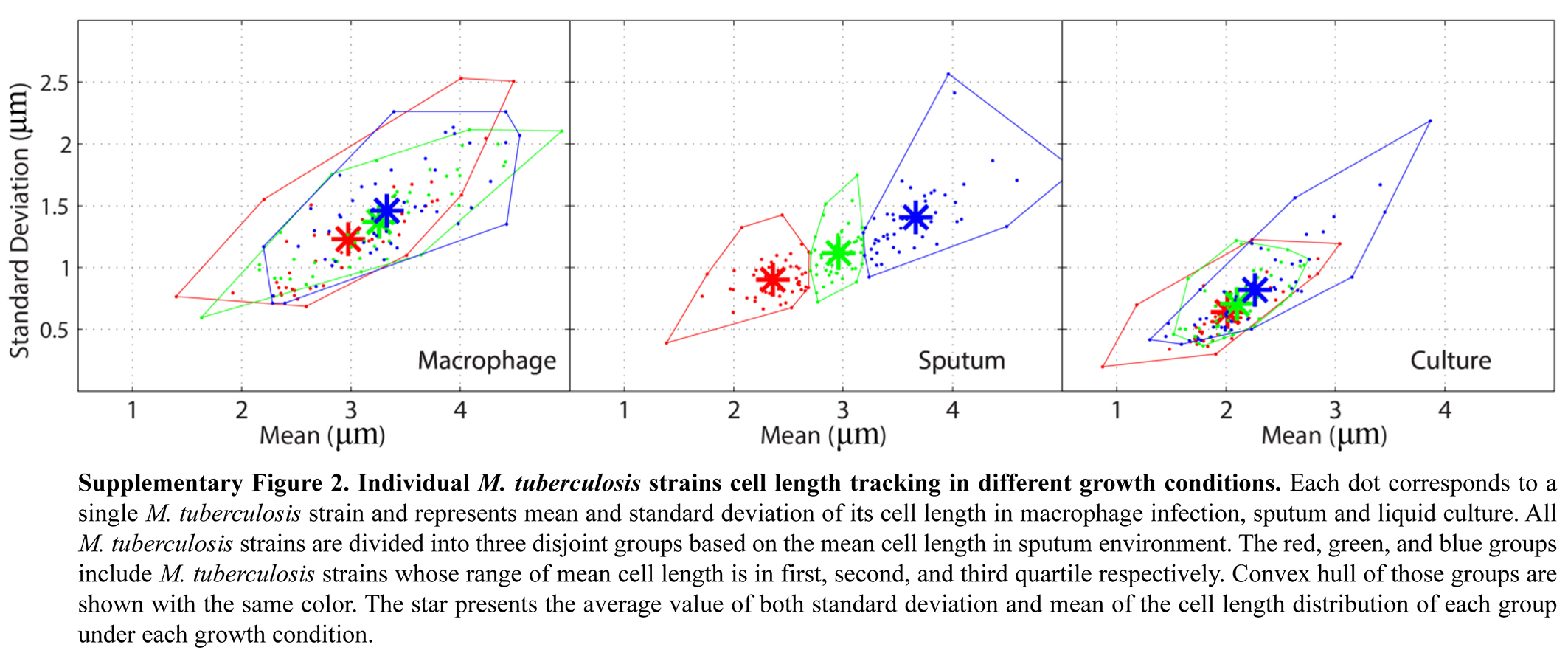

Supplement: Supplementary file 3 [file Image_2.TIF]

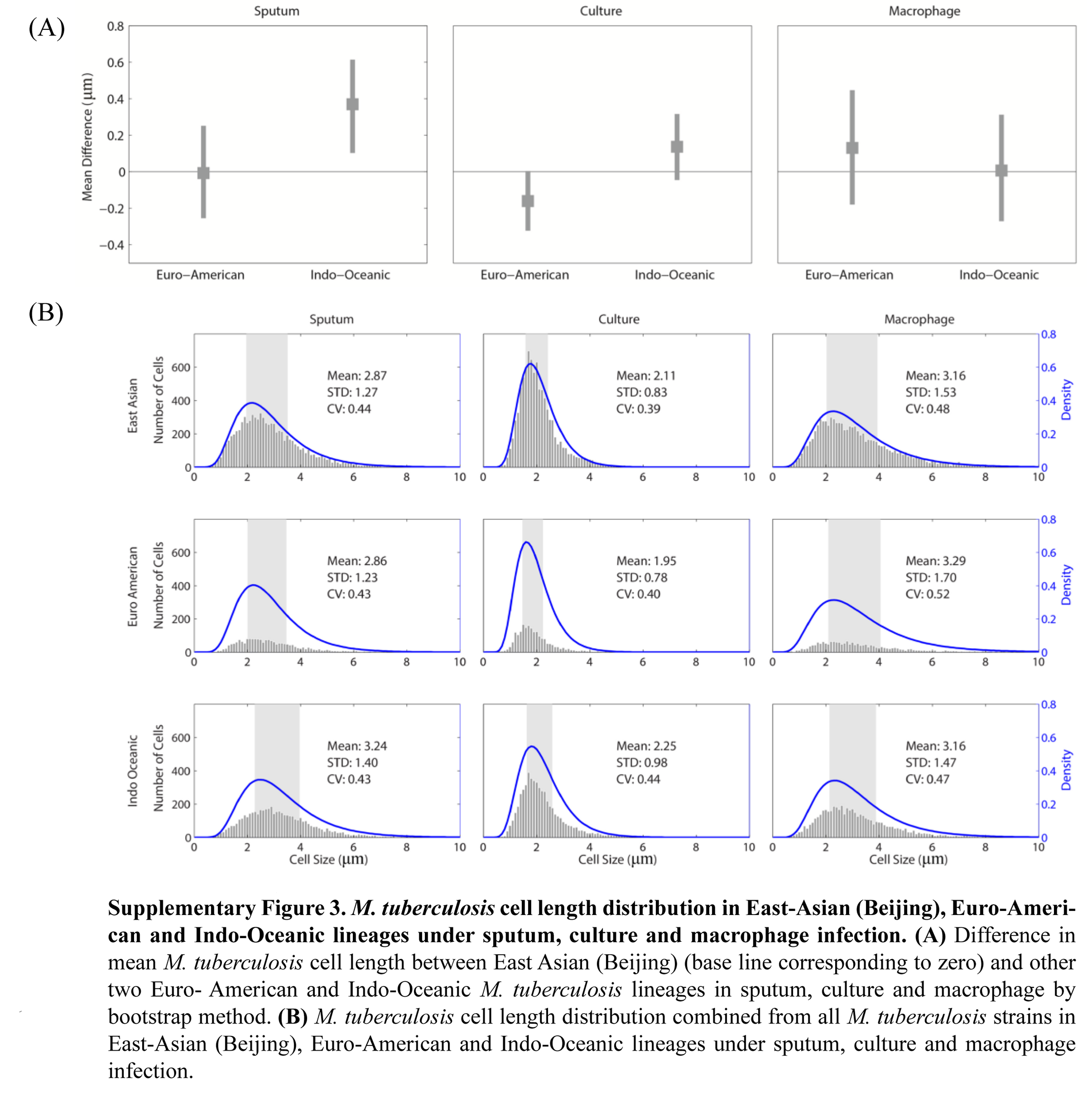

Supplement: Supplementary file 4 [file Image_3.TIF]

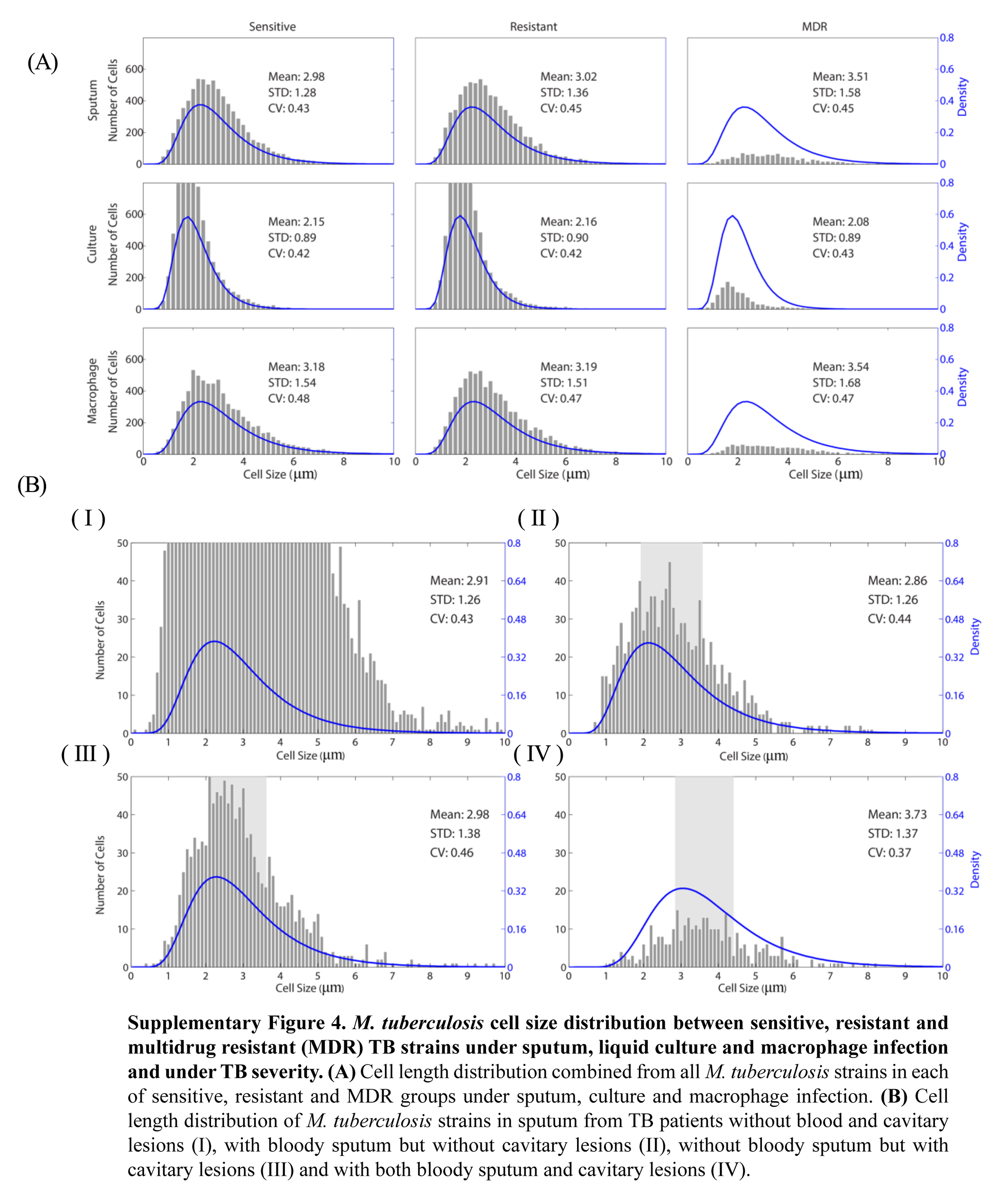

Supplement: Supplementary file 5 [file Image_4.TIF]

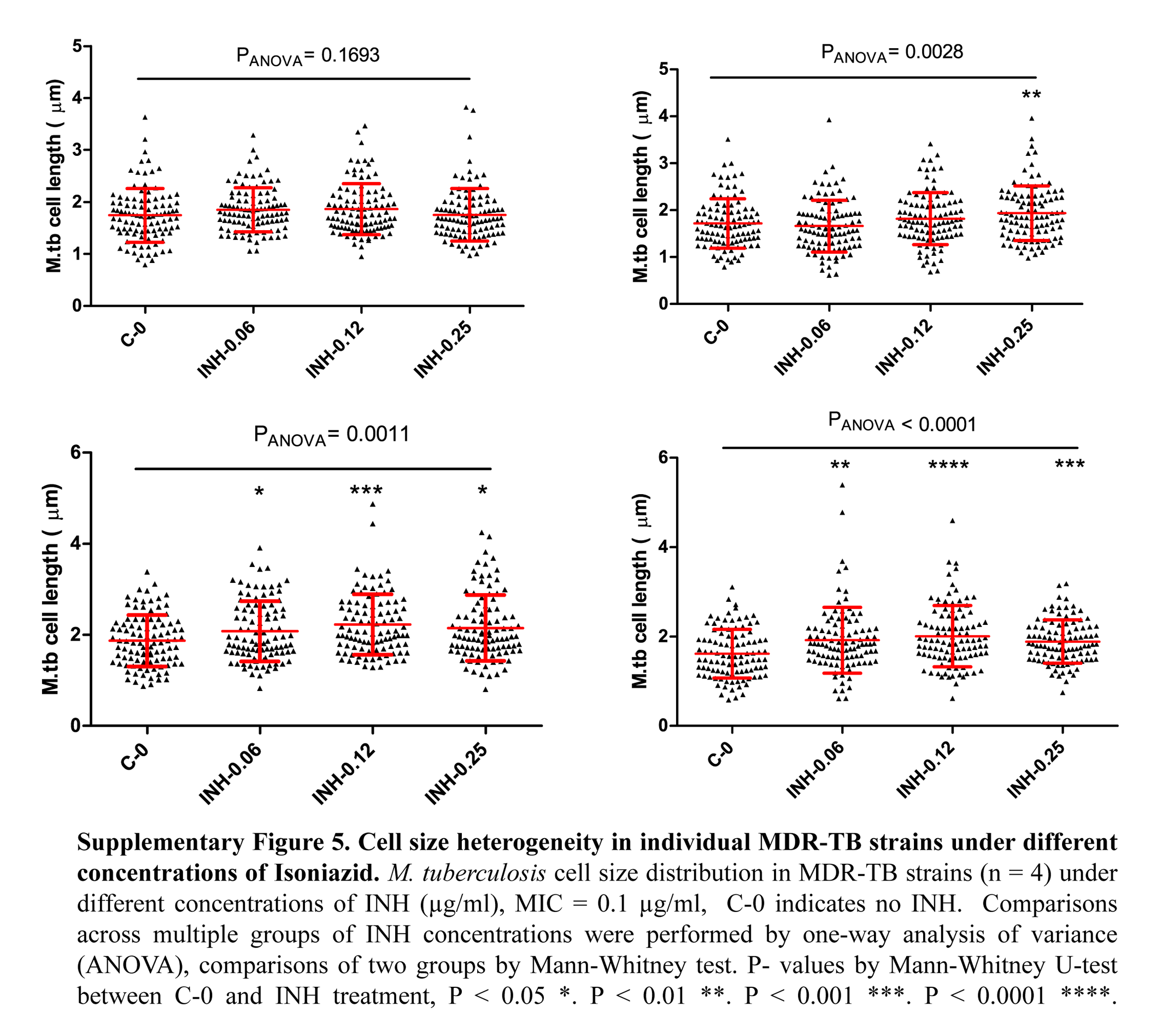

Supplement: Supplementary file 6 [file Image_5.TIF]
